# Chronic kidney disease prevalence in asymptomatic patients with risk factors—usefulness of serum cystatin C: a cross-sectional study

**DOI:** 10.1097/j.pbj.0000000000000233

**Published:** 2023-12-13

**Authors:** Mariela N. Avila, María C. Luciardi, Ana V. Oldano, Mariano N. Aleman, Rossana C. Pérez Aguilar

**Affiliations:** a Cátedra de Bioquímica Clínica III, Instituto de Bioquímica Aplicada, Facultad de Bioquímica, Universidad Nacional de Tucumán, Argentina; b Cátedra de Práctica Profesional, Instituto de Bioquímica Aplicada, Facultad de Bioquímica, Universidad Nacional de Tucumán, Argentina

**Keywords:** chronic kidney disease, CysC, overweight

## Abstract

**Background:**

Chronic kidney disease is recognized as a worldwide public health problem, particularly within an increasing prevalence of obesity, diabetes mellitus, and hypertension. This disease affects more than 13% of the world's population and is increasing. Further biochemical assessment with new biomarkers, such as serum cystatin C (CysC), would improve patient care and disease control. The aim of this study was to detect chronic kidney disease (CKD) in asymptomatic subjects with risk factors and evaluate CysC as early biomarker of renal damage and accurate test to estimation glomerular filtration (GF).

**Methods:**

This observational analytic and cross-sectional design included 195 patients of both sexes. A full clinical evaluation included height, weight, waist circumference, body mass index (BMI), blood pressure (BP), and family history of disease. Renal function was evaluated through serum creatinine (SCrea), serum CysC, urinary albumin, and urinary creatinine. GF was calculated using CKD-EPI creatinine (CKD-EPI Crea) and CKD-EPI creatinine-cystatin C equations (CKD-EPI Crea-CysC).

**Results:**

Renal injury showed 24% of patients with albuminuria; 18% of them were categorized as A2 and 6% as A3. Therefore, 73% had no progression risk (baseline risk), 20% moderate risk, and 7% high risk. Among analyzed groups, significant differences were found in BMI, BP, Screa, CysC, CKD-EPI Crea, and CKD-EPI Crea-CysC. Overweight population was analyzed by assessing CysC and calculating CKD-EPI Crea-CysC, showing an important change with respect to the general population.

**Conclusion:**

Combined CysC and Crea measurement provides incremental improvement in predicting measured GF.

## Introduction

Chronic kidney disease (CKD) is recognized as a worldwide public health problem with multiple etiologies, characterized by the progressive and silent loss of structurer and/or renal function. The global prevalence of CKD is more than 13% and has increased substantially in recent years, driven primarily by population growth and aging.^1^ In Argentina, according to the Second National Nutrition and Health Survey (2018–2019), 12.7% of adult population has CKD.^2^ Nevertheless, owing to the kidney's functional reserve, CKD is often asymptomatic or paucisymptomatic in its early stages and more than 10% of those subjects are not aware of its existence; for this reason, it has been called the silent epidemic.^3^

CKD was defined as decreased kidney function shown by estimated glomerular filtration (eGF) less than 60 mL/min per 1.73 m^2^ and/or evidence of kidney damage markers, at least for 3 months, regardless of the underlying cause. To manage CKD and provide better care for patients, CKD classification was developed by the international Kidney Disease Outcomes Quality Initiative guideline group Kidney Disease Improving Global Outcomes–2012 (KDIGO-2012) and National Institute of Health and Care Excellence.^4,5^

Thereby, patients with early-onset disease, stage 1–2, have normal or slightly reduced eGF levels (>90 to 60 mL/min/1.73 m^2^). Subjects with CKD stage 3a–3b have mild to moderately decreased eGF levels (59–45 and 45–30 mL/min/1.73 m^2^, respectively). The severely reduced eGF levels from stage 4 to 5 (29–15 and to <15 mL/min/1.73 m^2^, respectively) are indicative of advanced stages of disease and kidney failure. Stratification also comprises three categories of albuminuria. Patients with urinary albumin to urinary creatinine ratio (ACR) of 3–30 mg/g maximum have albuminuria and moderate risk of adverse outcomes. Those with ACR greater than 30 mg/g are at serious risk of developing adverse events.^1^

Albuminuria categories and eGF independently predict adverse outcomes for CKD, but combination of both further increases this risk. In this way, CKD classification helps clinicians to make accurate assessments of severity and other complications, which contributes to management and follow-up of patients with CKD.^1,6^

CKD is considered as a model of continuous progression, so risk factors identification allows its suspicion, early diagnosis in silent or asymptomatic stages, slowing or preventing its progression. CKD risk factors include personal and family history of kidney diseases, high blood pressure (BP), diabetes mellitus, obesity, and smoking.^7^ It was reported that obesity is associated with increased risk for CKD. This risk is also present in metabolically balanced overweight subjects, suggesting that obesity itself contributes to CKD independently of metabolic syndrome.^7,8^ However, there is limited information about the first stage of CKD prevalence in patients asymptomatic from Tucumán and the risk factors associated.

Cystatin C (CysC), a 13-kDa cysteine proteinase inhibitor protein, is normally produced by all nucleated cells at a constant rate and is filtered free by the kidney with near-complete reabsorption and catabolism in the proximal tubule and no significant urinary excretion. CysC has been proposed as a better endogenous biomarker of renal dysfunction, less influenced by age, sex, race, muscle mass, or body weight and useful for assessing GF and identifying subclinical renal disease with mild functional impairment.^9,10^

In this context and supported by the fact that one of ten subjects presents CKD at some stage of progression unknowingly, this study proposes to detect asymptomatic CKD in subjects with risk factors and evaluate the contribution of CysC as an early and accurate biomarker of function and renal damage.

## Methods

### Study design/patient selection

This observational analytic and cross-sectional design included 195 patients of both sexes, registered from different public hospitals in Tucumán (Argentina) and referred and evaluated at biochemistry, chemistry, and pharmacy faculty laboratory (National University of Tucumán) in March 2020 before COVID-19 social isolation.

Inclusion criteria were as follows: asymptomatic patients older than 18 years who attended public hospitals for routine studies and who agreed to participate in the Kidney Health Campaign. Exclusion criteria were as follows: patients with renal damage, such as persistent proteinuria, systemic infections, or known renal diseases or history of antibiotic treatment in previous month.

All patients underwent a full clinical evaluation, including height, weight, waist circumference (WC), body mass index (BMI), BP, history of kidney disease (HKD), urinary tract disease (UD), family history of kidney disease (FHKD), and family history of diabetes and/or cardiovascular diseases (CVD).

### Measurements and definitions

Weight and height were measured by a mechanical adult scale (Rome BPP-S w/Altimeter, Hijos de Francisco DINO S.R.L., Rosario, Argentina), with light clothing and no shoes and with ankles together, relaxed shoulders, and both arms at the sides of the bodies. BMI was estimated using the Quetelet index (weight/size^2^). WC was measured with an anthropometric tape measure (Lufkin W606PM, New York, NY) at the site of maximum circumference midway between the lower ribs and the anterior superior iliac spine. BP was averaged from two seated measurements using an automatic monitor (Omron Healthcare Co., HEM-7120, China).^11^

According to the World Health Organization (WHO), overweight and obesity are defined as BMI values equal to or higher than 25 and 30 kg/m^2^, respectively.^12^ Arterial hypertension was defined as BP ≥130/85 mm Hg and/or current use of antihypertensive drugs (cutoff values established by International Diabetes Federation and the American Heart Association/National Heart, Lung, and Blood Institute).^13^

### Biochemical parameters

First morning urine was collected by midstream technique, and blood samples were obtained between 8 and 10 am, by venipuncture, after nighttime fasting. Serum was isolated by centrifugation at 3500 rpm for 15 min and analyzed on the same day. Renal function was evaluated through serum creatinine (SCrea) and serum CysC levels. SCrea was determined by kinetic and enzymatic methods, traceable to reference method (Creatinine Kinetic AA and Creatinine Enzymatic AA, Wiener Lab) and CysC by immunoturbidimetric method (Cystatin C Turbitest AA, Wiener Lab). GF was estimated using Chronic Kidney Disease Epidemiology Collaboration equations (CKD-EPI): CKD-EPI creatinine (CKD-EPI Crea) and CKD-EPI creatinine-cystatin C equations (CKD-EPI Crea-CysC).^14^ Initially, the progression of CKD was performed with the measurement of albumin-to-creatinine ratio (UACR) and CKD-EPI Crea, according to KDIGO, 2012.^7^ In addition to excess weight population, CKD risk category (G1-G5) was compared with CKD-EPI Crea and CKD-EPI Crea-CysC calculation.

Urinary albumin (UA) and creatinine (UCrea) were measured by immunoturbidimetric and kinetic methods, respectively (Microalbumin Turbitest AA and Creatinine Kinetic AA, Wiener Lab). Albuminuria was reported as UACR and defined as 30–299 mg/g. Biochemical evaluation was complemented with a complete blood count, urea and glucose, total proteins, and serum albumin (Reagents AA, Wiener Lab).

### Statistical analysis

Statistical analysis was performed using the IBM SPSS Statistics, version 25.0 (IBM Co., Armonk, NY). The Kolmogorov-Smirnov test was used to determine quantitative variables distribution. All data were expressed as frequency and percentage for categorical data and mean ± standard deviation. Differences in study participants' characteristics were compared across subgroups with the chi-square test for categorical variables and the *t* test as appropriate. Differences in mean levels of continuous variables between subgroups were determined using one-way analysis of variance (ANOVA) with the Tukey post hoc test as appropriate for multiple comparisons. The statistical power calculation was performed using the G*Power software version 3.1.9.6 (Franz Faul, University Kiel, Germany). A *P* value <.05 was considered significant.

### Ethical statement

The study was approved by Tucuman University Investigation Ethics Committee (CEI, Tucumán-Argentina, CEI PIUNT, October 18th, 2017); written informed consent for participation was obtained from all the patients.

## Results

Clinical and metabolic characteristics of evaluated subjects are shown in Table 1. The mean age was 54 ± 12 years. Around two-thirds of population was overweight, and half had hypertension and FHKD. Only one-third had diabetes mellitus and family history of this disease. Furthermore, one-fifth of the participants were smokers and reported UD.

**Table 1.** Clinical characteristics of the groups studied.

N	195
Male/female	40/155
Age (years)	54 ± 12
Weight (kg)	78.72 ± 18.9
Height (m)	1.50 ± 0.76
BMI (kg/m^2^)	30.54 ± 6.42
WC	
Male (cm)	102.72 ± 21.38
Female (cm)	99.03 ± 16.44
SBP (mmHg)	134 ± 21
DBP (mmHg)	79 ± 12
HBP	
No	88 (45.12%)
Yes	107 (54.88%)
Diabetes	
No	125 (64.10%)
Yes	70 (35.90%)
Overweight	
No	69 (35.40%)
Yes	126 (64.60%)
Smoker	
No	156 (80%)
Yes	39 (20%)
HKD	
No	131 (67.20%)
Yes	64 (32.80%)
UD	
No	158 (82.60%)
Yes	34 (17.40%)
FHKD	
No	102 (52.30%)
Yes	92 (47.70%)

All data are expressed as frequency for categorical data and mean ± standard deviation for numeric data.

BMI, body mass index; DBP, diastolic blood pressures; FHKD, family history of kidney disease; HBP, high blood pressure; HKD, history of kidney disease; SBP, systolic blood pressure; UD, urinary tract disease; WC, waist circumference.

Renal injury evidenced as UACR showed 24% of patients (n = 48) with albuminuria; 18% of them (n = 35) were categorized as A2 (albuminuria of 30 at 299 mg/g) and 6% (n = 13) as A3 (albuminuria >300 mg/g). According to KDIGO, 2012, measurement of UACR and CKD-EPI Crea allows diagnosis, prognosis, and staging of CKD progression.^4,7^

The results showed that 73% of studied subjects (n = 143) had no progression risk (BR: baseline risk, no renal disease, stages G1-A1 or G2-A1), 20% (n = 39) had moderate risk (MR, stages G1-A2 or G2-A2), and 7% (n = 13) high risk of progression (HR, G1-A3, G2-A3 or G3b-A1). The variance test was performed to study the differences between clinical and renal parameters of progression risk groups.

Among analyzed groups, significant differences were found in BMI, SBP, DBP, Screa, CysC, and CKD-EPI Crea-CysC (*P* value <.05). Patients with HR had higher BMI, SBP, and DBP compared with the BR group. Also was evidenced significantly higher SBP and DBP with respect to subjects with MR. Parameters of renal function showed Screa, CysC, and CKD-EPI Crea-CysC values significantly different in MR and HR vs. BR groups, but no significant differences were observed between MR and HR. Table 2.

**Table 2.** Metabolic and renal parameters according to CKD risk

	BR (n = 143)	MR (n = 39)	HR (n = 13)	*P*	β
BMI (kg/m^2^)	29.63 ± 5.17*	32.04 ± 7.67	35.91 ± 10.69*	.001	0.95
WC (cm)	98.68 ± 15.90	101.37 ± 22.00	109.54 ± 18.25	.87	0.95
SBP (mmHg)	131.00 ± 18.00*	137.00 ± 21.00†	163.00 ± 27.00*,†	.000	0.95
DBP (mmHg)	78.00 ± 11.00*	79.00 ± 13.00†	89.00 ± 12.00*,†	.005	0.96
SCrea (E) (mg/dL)	0.63 ± 0.14‡,*	0.72 ± 0.22‡	0.79 ± 0.26*	.012	0.95
CysC (mg/L)	0.93 ± 0.26‡,*	1.23 ± 0.34‡	1.32 ± 0.43*	.001	0.95
CKD-EPI Crea (mL/min)	103.87 ± 14.96‡,*	95.79 ± 24.96‡	91.23 ± 22.09*	.005	0.97
CKD-EPI Crea-CysC (mL/min)	104.25 ± 23.46‡,*	76.87 ± 22.26‡	74.23 ± 25.55*	.000	0.96

One-way ANOVA test (significant *P* < .05).

BMI, body mass index; BR, baseline risk; CKD, chronic kidney disease; CKD-EPI Crea, Chronic Kidney Disease Epidemiology Collaboration equation using serum creatinine by enzymatic method; CKD-EPI CysC-Crea, Chronic Kidney Disease Epidemiology Collaboration equation using serum creatinine by enzymatic method and cystatin C; CysC, serum cystatin C; DBP, diastolic blood pressures; HR, high risk; MR, moderate risk; SBP, systolic blood pressure; SCrea (E), serum creatinine by enzymatic method; WC, waist circumference

* *Post hoc* analysis revealed significant difference between BR and HR.

† *Post hoc* analysis revealed significant difference between MR and HR.

‡ *Post hoc* analysis revealed significant difference between BR and MR.

**Figure 1.**
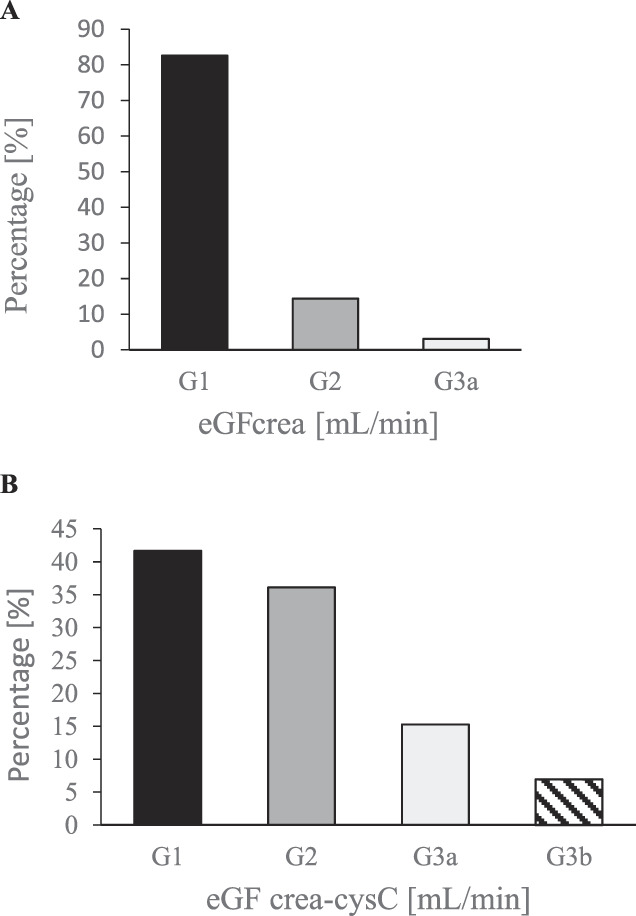
Bar charts showing percentages of subjects according to eGFR categories (G1-G5). (A) CKD risk categories based on eGF using CKD-Epi creatinine equations (G1-G5); (B) CKD risk categories based on eGF using CKD-Epi creatinine-cystatine C equations (G1-G5). The numbers on the bars represent the percentage.

To evidence underlying effects of excess weight on renal function, overweight population (n = 126) was analyzed by assessing CysC and calculating CKD-EPI Crea-CysC. Initial classification (with CKD-EPI Crea) of excess weight patients showed that 83% (n = 104) belonged to G1 category, 14% (n = 18) to G2, and 3% (n = 4) to G3a. When classification was performed with CKD-EPI Crea-CysC, significant changes were observed in FG categories, reclassifying as G1: 42% (n = 53), G2: 36% (n = 45), G3a: 15% (n = 19), and G3b: 7% (n = 9). This suggests that excess weight has an underlying effect on renal function (Fig. 1A-1B). The use of CysC associated with SCrea as endogenous marker recategorizes CKD risk progression, showing a greater number of patients with HR.

## Discussion

In recent decades, driven by demographic growth, population aging, and global prevalence, the incidence of CKD-related complications such as heart disease have increased considerably, becoming a major public health problem worldwide.^1^ In our country, estimated global CKD prevalence was 12.7%, but these data are only for patients with advanced stage disease requiring renal function replacement; nevertheless 1 of 10 adults have some form of CKD and are unaware they have it.^2,3^

A conceptual model for development, progression, and complications of CKD was proposed, which recognizes risk factors that allow its suspicion, early diagnosis, and staging. This model identifies earlier stages, considered potentially reversible, and links them to kidney failure at the end stage of CKD.^14^ According to this, kidney failure is preceded by kidney damage that may be insensitive and nonspecific and a decrease in glomerular filtration.^15^ The presence of risk factors allows suspecting asymptomatic CKD and timely detection of kidney damage.

Identification and staging of patients at risk of kidney disease is an essential part of clinical nephrology. This is achieved by assessment of GFR, which is considered the best marker of renal function in health and disease. For long time, urinary inulin clearance over a 24-hour period has been considered as the reference method for GF rate measurement and evaluation. However, its use is difficult to apply in routine clinical practice and research purpose; its invasiveness, complexity, and costs limit the availability of this technique.^16^ Recently, this method has been taken off the market in France for safety reasons. Two other alternatives have emerged, in Europe, iohexol clearance is widely used and in the United States, iothalamate clearances.^17,18^ Therefore, their uses are limited to circumstances where an accurate determination of the GF rate is required. These include evaluations of living kidney donor, CKD progression in patients receiving contrast media, dose adjustment of nephrotoxic drugs, and patients in clinical protocols, or they are used as a guide therapeutic decision based on rate filtration, such as the initiation of dialysis in doubtful situations.^19^

New advances have been made in the last 20 years regarding the use of large databases and statistical methods to estimate the GF rate using endogenous biomarkers. Nowadays, eGFR is recommended by clinical practice guidelines and regulatory agencies in the routine assessment of renal function.^19,20^ In fact, in our country, scientific and professional societies recommend evaluating renal function through eGFR based on serum creatinine and suggest the use of the equations developed by the Chronic Kidney Disease Epidemiology Collaboration (CKD-EPI) because of their precision.^21^

Although creatinine is the most common marker of endogenous filtration to estimate GFR, serum CysC has established itself as an early and accurate biomarker of CKD especially useful in patients in whom creatinine is an inadequate marker.^15^ In this context, the aim of this work was to detect and determine CKD prevalence in asymptomatic subjects with risk factors and analyze CysC contribution as early biomarker of renal damage and precise test for evaluating GF.

This study showed that approximately a quarter of the studied population had kidney damage. In concordance with our results, a screening program that included 400 subjects asymptomatic with CKD risk and never diagnosed with kidney disease revealed that 18% of them had renal damage. However, prevalence increased to 27.4% when the age-matched population was considered.^22^ Li et al, in a study with 1811 patients, obtained similar results, showing CKD prevalence of 24% in adult population apparently healthy.^23^

Overweight was the most prevalent risk factor assessed in our population (64.6%). These results agree with the Fourth National Survey of Risk Factors published in 2018, showing 61.6% of adults with excess weight (36.2% overweight and 25.4% obese).^2^ The first Health at a Glance publication to cover the Latin America and the Caribbean region prepared jointly by Organization for Economic Co-operation and Development (OECD) and the World Bank showed similar results: In OECD countries, 63% of men and 52% of women are overweight; in Mexico and Chile, over 75% of their female population is overweight, while Chile leads the region with 74% of its male population being overweight followed by Mexico (70%) and Argentina (66%).^24^

The analysis of the relationship between overweight and CKD risk showed a significant rise in BMI among the population at risk compared with the nonrisk group. Similar results were found in a Japanese research of 3087 patients, showing that increased BMI was associated with an increased risk of CKD progression in the range of BMI ≥25.^25^ Nevertheless, the data were not conclusive. An investigation in Guatemala revealed that 4.0% of the study population had CKD, and the most important factors associated with risk of disease were a diabetes history or hypertension and low weight (BMI <18.5).^26^

It is well known that overweight and hypertension contributes directly toward cardiovascular disease and CKD.^27^ In fact, increased fat may predispose to CKD because of the link directly with obesity glomerulopathy and indirectly with the complications associated with it, such as atherosclerosis, hypertension, and type 2 diabetes.^28^ Our research found that hypertension was the second most important risk factor (54.88%). According to Geldsetzer et al,^29^ these values are well above of prevalence reported for other Latin American populations (Chile, Peru, Brazil, Mexico, and others). However, a cross-sectional study conducted in Oxfordshire, United Kingdom, which recruited 3207 subjects, found a hypertension prevalence of 62% in apparently healthy subjects with CKD suspect.^30^ With respect to hypertension, we observed that BR and MR groups were significantly different from HR. A recent review showed an association between hypertension grade and progressive risk of developing CKD when subjects are stratified according to hypertension severity. It also concluded that the CKD risk increases with the hypertension grade.^31^

Chronic diseases, muscle mass alterations, and advanced age affect serum creatinine.^32^ BMI also affects creatinine excretion and interpretation UACR. This implies that both GF measurement and estimation, based on creatinine, would lead to a significant underestimation bias.^33^ In this context, the eGF could be more accurate, using another endogenous biomarker such as CysC. This cysteine proteinase inhibitor protein has been established as an early and accurate biomarker of CKD, particularly in patients for whom creatinine is an inadequate marker.^34^ Therefore, its levels are much less affected by patient characteristics such as sex, age, body size and composition, and nutritional status. It is highlighted that urinary iothalamate clearance, test of measured FG, is the basis for the CKD-EPI equations using creatinine and CysC that are recommended by current guidelines.^35,36^

Owing to the high overweight prevalence and following this approach, a restratification in this group was performed using CKD-EPI Crea-CysC equation. Our data suggest that the equation based on Crea and CysC in combination would be useful in the overweight population by achieving a reclassification of individuals in advanced CKD stages. A Japanese study where the performance of the same equations was tested reported similar results and concluded that using CKD-EPI equations, CKD-EPI Crea-CysC is less biased, more precise, and more accurate than CKD-EPI Crea and CKD-EPI CysC.^37^ Thus, combined CysC and Crea measurement provides incremental improvement in predicting measured GF. It would, though, be associated with substantial initial costs to the health care system.^34,38^

The relevant finding of this research was to provide data on CKD prevalence in asymptomatic subjects from Tucumán, where the information is still limited; similarly, to analyze CysC and CKD-EPI Crea-CysC contribution in the evaluation of apparently healthy patients with excess weight as the most prevalent risk factor. The addition of CysC to Crea was shown to improve the accuracy of GF estimation in overweight patients. Thus, combining both filtration markers is more accurate and would support better clinical decisions than either marker alone.^39^

This research has some limitations; it is a preliminary cross-sectional design with a relatively small sample size; so it only allows associations. On the other hand, this study was financed with subsidies from the National University of Tucumán, and the analysis of the economic cost to the health system was not performed. The benefits of CysC use according to different age groups should also be considered. In this context, and although the general use of CysC to CKD screening requires further study, there are patient subgroups for which CysC has been shown to be a superior biomarker, and it should be used to accurately assess FR.

## Funding

This research was supported by Consejo de Investigaciones de la Universidad Nacional de Tucumán (CIUNT) and Proyecto Extensión de la Secretaría de Políticas Universitaria, Argentina; grants to Rossana Cristina Pérez Aguilar, PhD.

## Conflict of interest

The authors did not report any conflict of interest.
